# Light up the mitochondria: Smart tungstate-oligosaccharide nanoplatform orchestrates mitochondrial transfer from M2 macrophages to restore endothelial function for adaptive diabetic wound regeneration

**DOI:** 10.1016/j.mtbio.2025.102196

**Published:** 2025-08-14

**Authors:** Xiuhong Huang, Ziling Lin, Mingshu Ruan, Peizhen Huang, Hongmei Ding, Hao Pan, Jiahui Cao, Chunmei Ma, Qianhao Zhao, Wenping Guo, Keke Wu, Chongkai Fang, Aijun Liu, Liqin Zheng

**Affiliations:** a School of Basic Medical Sciences, Guangzhou University of Chinese Medicine, Guangzhou, 510006, China; b Department of Hand Surgery and Wound Repair, The First Affiliated Hospital of Guangzhou University of Chinese Medicine, Guangzhou, 510405, China; c International Collage, Guangzhou University of Chinese Medicine, Guangzhou, 510405, China; d School of Biomedical Engineering, Affiliated Cancer Hospital & Institute, Guangzhou Medical University, Guangzhou, 511436, China; e Science and Technology Innovation Center, Guangzhou University of Chinese Medicine, Guangzhou, 510405, China

**Keywords:** Mitochondria transfer, Tungstate-oligosaccharides nanoplatform, Macrophage, Angiogenesis, Diabetic wound

## Abstract

Diabetic wound (DW) complications, driven by persistent oxidative stress, unresolved inflammation, and vascular dysfunction, present a critical clinical challenge. Given mitochondria's pivotal role in inflammatory regulation, intercellular mitochondrial transfer emerges as a promising therapeutic target for DW management. In this study, we engineered a ROS/glucose/pH-triple responsive nanoplatform (WOC) via coordination-driven assembly of tungstate anions (WO_4_^2−^) and chitosan oligosaccharide (COS) to synchronize immunomodulation and angiogenesis for adaptive DW regeneration. The WOC platform demonstrated glucose/pH-triggered release of bioactive components with moderate ROS scavenging capacity, enabling real-time monitoring via visible colorimetric transition. By enhancing mitochondrial bioenergetics, WOC polarized macrophages to M2 phenotype and orchestrated vesicles-dependent mitochondrial transfer to injured endothelial cells, restoring vascular function through upregulated angiogenesis genes, enhanced migration, and tube formation. In diabetic rat models, WOC accelerated wound closure evidently, resolving inflammation and promoting scarless regeneration via balanced collagen deposition. This work establishes mitochondrial transfer as a promising strategy, offering a tunable nanotherapeutic approach to recalibrate cellular cross-talk and microenvironment dynamics in DW healing.

## Graphical abstract

**Figure:**
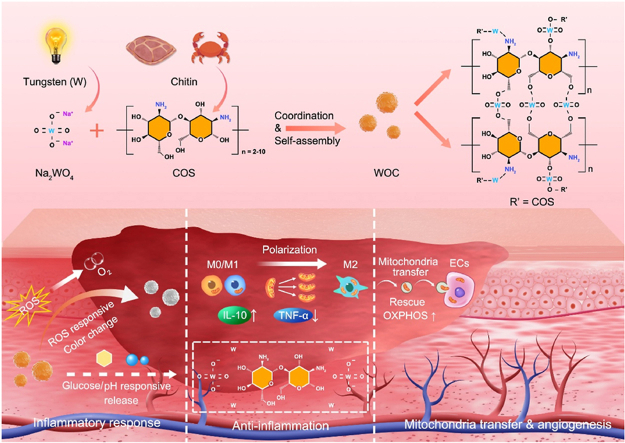

## 1 Introduction

Diabetic wounds (DW) affect 18.6 million patients globally, with 15–20 % requiring amputations - making diabetes the leading cause of non-traumatic limb loss [1]. Post-amputation mortality exceeds many cancers (5-year survival <50 %) [2], highlighting the urgent need for innovative therapeutic strategies targeting DW's core pathomechanisms: chronic hyperglycemia-induced hypoxia, oxidative stress, vascular dysfunction, and persistent inflammation [[3], [4], [5]]. This has spurred preclinical development of anti-inflammatory/angiogenic functional dressings [[6], [7], [8]].

Macrophage plasticity governs wound repair dynamics: M1 phenotypes drive early inflammation via mitochondrial reactive oxygen species (ROS)-mediated pathogen clearance, whereas M2 phenotypes orchestrate tissue remodeling through oxidative phosphorylation (OXPHOS)-dependent mechanisms [[9], [10], [11]]. However, the hyperglycemia, hypoxia, and oxidative stress microenvironment locks macrophages into a pro-inflammatory M1 state, impairing their repolarization to regenerative M2 phenotypes even under interleukin-4 (IL-4) stimulation [12]. This metabolic paralysis arises from disrupted mitochondrial bioenergetics, as M1 polarization relies on aerobic glycolysis, while M2 functionality requires intact OXPHOS [[13], [14], [15], [16]]. Consequently, restoring mitochondrial OXPHOS in macrophages represents a linchpin for resolving inflammation and enabling angiogenesis in DW.

Angiogenic failure, driven by endothelial dysfunction, further exacerbates DW chronicity. Intercellular mitochondrial transfer, which is a natural rescue mechanism where healthy cells donate mitochondria to compromised counterparts, has emerged as a potent strategy for restoring OXPHOS, mitigating oxidative damage, and rejuvenating cellular function [17,18]. Notably, M2 macrophages not only exhibit pro-angiogenic paracrine effects but also serve as mitochondrial donors, suggesting their dual role in vascular repair [[19], [20], [21]]. Capitalizing on this biology, we posit that targeted mitochondrial transfer from M2 macrophages to endothelial cells could break the vicious cycle of inflammation and poor vascularization in DW.

Metal ions, with their inherent positive charge and redox activity, offer unique advantages for mitochondrial targeting given the organelle's electronegative stress microenvironment [[22], [23], [24]]. Tungsten-based nanoplatform (TNs) have gained traction in biomedicine due to their tunable catalytic properties, structural diversity, and applications in biosensing, antimicrobial therapy, and drug delivery [25,26]. TNs encompass a wide range of nanoscale multifunctional materials primarily composed of tungsten (W) and its various compounds, such as tungsten oxide (WO_x_), tungsten sulfide (WS_x_), tungsten carbide (WC_x_), and tungsten nitride (WN_x_) [27]. Biochemically, W-containing oxidoreductase catalyzes the conversion of toxic aldehydes to acids via electron bifurcation, coupling aldehyde oxidation with nicotinamide adenine dinucleotide^+^ (NAD^+^) and ferredoxin reduction [28], yet its potential in mitochondrial bioenergetics regulation remains unexplored. Chitosan oligosaccharide (COS), a natural degradation product of chitin with anti-inflammatory and immunomodulatory properties [29], synergistically enhances M2 polarization by boosting OXPHOS and anti-inflammatory cytokine secretion [19,[30], [31], [32]]. Despite these individual merits, the strategic integration of tungstate's catalytic plasticity and COS's immunomodulatory capacity remains unexplored for engineering mitochondria-targeted nanotherapeutics in diabetic wound management.

**Scheme 1:**
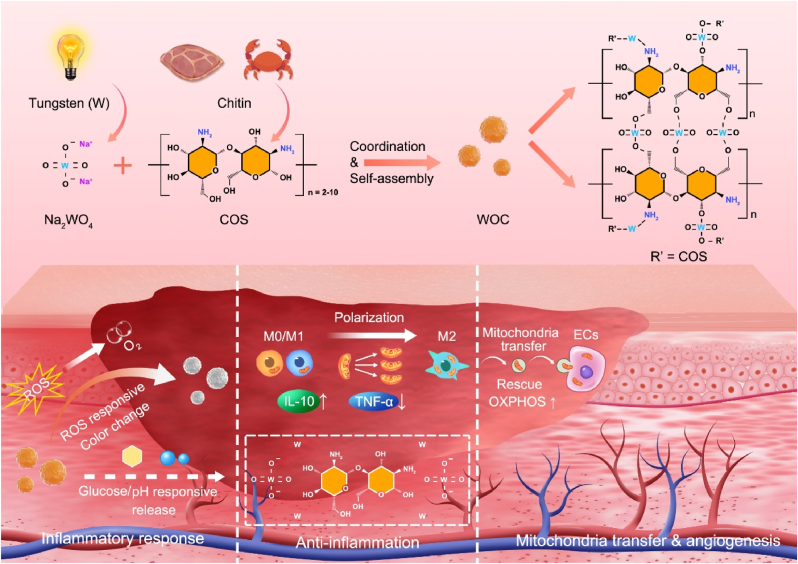
The WOC nanoplatform orchestrates multi-stage diabetic wound (DW) repair through three synergistic mechanisms: (1) Microenvironment-Responsive Bioactivity: Exhibiting ROS-triggered colorimetric signaling and glucose/pH-dependent release of bioactive W element and COS, enabling spatiotemporal drug delivery; (2) Metabolic Reprogramming: WOC-enhanced mitochondrial bioenergetics drive macrophage M2 polarization, establishing an anti-inflammatory milieu; (3) Vascular Restoration: Vesicles-mediated mitochondrial transfer from M2 macrophages to endothelial cells rescues OXPHOS and angiogenesis, while suppressing hypertrophic scar. By synchronizing immunomodulation, mitochondrial transfer, and vascular repair, WOC achieves faster wound closure.

Herein, we report a glucose/pH-responsive nanoplatform (denoted as WOC) through coordination-driven assembly of WO_4_^2−^ and COS, designed to synchronize immunomodulation with mitochondrial transfer for adaptive DW healing. The WOC system enables spatiotemporal release of bioactive WO_4_^2−^ and COS in response to DW-specific hyperglycemia and alkalosis, coupled with self-reporting colorimetric ROS scavenging; metabolic reprogramming of macrophages via OXPHOS amplification, driving M2 polarization; directed mitochondrial transfer from M2 macrophages to injured endothelial cells, rescuing viability, migration, and tube formation under oxidative stress; angiogenic gene activation through mitochondrial functional restoration. By integrating mechanistic studies and preclinical validation, this work establishes a novel approach for intracellular mitochondrial therapy in DW management, bridging immunometabolic regulation with cellular cross-talk engineering (Scheme 1).

## 2 Materials and methods

### 2.1 Materials and reagents

Sodium tungstate dihydrate (Na_2_WO_4_·2H_2_O, AR 99.5 %) was procured from Macklin Reagent (Shanghai, China). Chitosan oligosaccharide (COS, average molecular weight Mw ≤ 1000, 2 ≤ DP ≤ 10) was purchased from Dalian GlycoBio Company (Liaoning, China). Macrophages cell line (RAW264.7, ATCC Number: TIB-71) and human umbilical vein endothelial cell (HUVECs) were sourced from the Cell Bank of Type Culture Collection of the Chinese Academy of Sciences (Shanghai, China, cat. no. # SCSP-5285). Ultrapure deionized water was used for all aqueous solutions.

### 2.2 Synthesis of WOC nanoplatform

1.0 g of COS was fully dissolved in 100 mL of deionized water under continuous magnetic stirring at 25 °C. Subsequently, 100 mL of Na_2_WO_4_·2H_2_O aqueous solution (5 mg mL^−1^) was added dropwise to the COS solution, followed by vigorous stirring for 24 h at ambient temperature to facilitate the coordination between WO_4_^2−^ anions and the hydroxyl/amino groups of COS. The resulting mixture was then centrifuged at 10000 rpm for 5 min to collect the coordination complex, which was subsequently washed five times with deionized water via repeated centrifugation cycles (10000 rpm, 5 min each) to remove unreacted precursors and impurities. Finally, the purified WOC product was lyophilized at −80 °C for 24 h to obtain a stable powder for further characterization and application.

### 2.3 Material characterization

The morphological features were analyzed via field-emission scanning electron microscopy (SEM; ULTRA 55, Zeiss, Germany) and high-resolution transmission electron microscopy (TEM; JEM-F200, JEOL, Japan), with elemental distribution mapping performed using an energy-dispersive X-ray spectroscopy (EDS) detector integrated into the TEM system. Crystalline structure was evaluated by X-ray diffraction (XRD; MiniFlex 600, Rigaku, Japan), while chemical bonding and functional groups were identified through Fourier-transform infrared spectroscopy (FTIR; Nicolet iS10, Thermo Scientific, USA). Thermal stability was assessed via thermogravimetric analysis (TGA; NETZSCH STA 449 F5, Germany) under a nitrogen atmosphere. Colloidal properties, including hydrodynamic diameter and surface charge, were quantified using dynamic light scattering (DLS; Nano ZS, Malvern Panalytical, USA) with a zeta potential analyzer.

### 2.4 Free radical scavenging activity and multi-stimuli responsive behavior

The antioxidant capacity of WOC was evaluated through 1,1-diphenyl-2-picryl hydrazyl (DPPH) and 2,2′-azinobis-3-ethylbenzthiazoline-6-sulphonate (ABTS^+^) radical scavenging assays. For the DPPH assay, WOC was incubated with a 0.2 mM ethanolic DPPH solution (1:1 v/v) for 30 min in the dark, and the absorbance at 517 nm was measured using UV–vis spectroscopy to calculate scavenging efficiency. Similarly, the ABTS^+^ assay involved reacting WOC with pre-generated ABTS^+^ radical cations, followed by absorbance measurement at 734 nm after 6 min.

To assess the glucose- and pH-responsive release kinetics, WOC samples were incubated in 0.1 M phosphate-buffered saline (PBS) at varying pH levels (5.5, 7.4, 8.5) and a 25 mM glucose solution (pH 7.4) under physiological conditions (37 °C, 100 rpm). At specified intervals (1, 3, 7 days), released W was quantified via inductively coupled plasma mass spectrometry, while COS content was determined fluorometrically using a microplate reader (λex = 250 nm, λem = 440 nm).

The ROS-responsive behavior of WOC was comprehensively characterized through nanozyme activity assays and redox-state analysis. Superoxide dismutase (SOD)-like activity was evaluated by monitoring nitroblue tetrazolium reduction inhibition, whereas catalase (CAT)-like functionality was assessed by time-dependent O_2_ generation using dissolved oxygen meter (5 mg WOC was added to 1 mL H_2_O_2_). Concurrently, oxygen microbubble-driven locomotion was observed and videoed under optical microscopy. Time-dependent colorimetric transitions of WOC in H_2_O_2_ were recorded using a digital camera, revealing a visible shift from brown to white over 7 days. To elucidate the redox mechanism, electron paramagnetic resonance (EPR) spectroscopy was employed before and after H_2_O_2_ exposure (880 mM, 6 h), demonstrating W valence transitions via g-factor analysis.

### 2.5 Cell culture

RAW264.7 cells were cultured in DMEM supplemented with 10 % fetal bovine serum (FBS), 100 U mL^−1^ of penicillin, and 100 μg mL^−1^ of streptomycin in a humidified atmosphere containing 5 % CO_2_ at 37 °C. HUVECs were cultured in basal medium supplemented with culture supplement and 10 % FBS, which were obtain from OriCell.

### 2.6 Macrophages polarization and mitochondrial function

Macrophages RAW264.7 were treated with IL-4 (20 ng mL^−1^), glutathione (GSH, 100 μg mL^−1^), and WOC (100 μg mL^−1^) for 3 days, then harvested for subsequent functional analysis. The cell viability of RAW264.7 was detected using the Cell Counting Kit-8 (CCK8) assay. Ultra-high-throughput protein microarray analysis was performed on supernatants from WOC-treated macrophages, followed by KEGG pathway enrichment to identify key inflammation-related signaling pathways. Flow cytometry quantified the expression ratio of surface markers CD206 (M2 phenotype) to CD80 (M1 phenotype) to evaluate polarization status. ELISA was employed to measure secretion levels of the anti-inflammatory cytokine IL-10 and pro-inflammatory cytokine TNF-α in cell supernatants. Mitochondrial membrane potential (MMP) was assessed using the JC-1 fluorescent probe, with fluorescence microscopy visualizing J-aggregate (red) and monomer (green) distribution, and a microplate reader quantifying red/green fluorescence intensity ratios. Intracellular ATP content was measured via a luciferase-based luminescence assay, while SOD and CAT activities were determined using WST-8 and UV absorption methods, respectively. Western blotting analyzed Nrf-2 and HO-1 protein expression to validate antioxidant pathway activation.

### 2.7 Macrophages mitochondrial biogenesis

Mitochondrial mass was first assessed using MitoTracker Deep Red FM fluorescent labeling, with fluorescence microscopy imaging and quantitative intensity analysis. qRT-PCR was performed to measure the expression of mitochondrial biogenesis-related genes (SIRT1, PGC-1α, UQCRC2, MTCO1, ATP5A), normalized to β-actin. TEM was employed to visualize ultrastructural mitochondrial morphology and quantify mitochondrial counts per cell.

### 2.8 Molecular docking analysis

The three-dimensional structure of COS (PubChem CID: 3086191) was retrieved in SDF format from the PubChem database (https://pubchem.ncbi.nlm.nih.gov/). Crystal structures of SIRT1 (PDB ID: 4I5I, resolution 2.5 Å) and PGC-1α (PDB ID: 8BF1, resolution 1.36 Å) were obtained from the RCSB Protein Data Bank (https://www.rcsb.org). Prior to docking, protein structures were preprocessed by removing crystallographic water molecules and adding polar hydrogen atoms, while the COS ligand was energy-minimized to optimize geometry. All structures were converted to PDBQT format to ensure docking compatibility. A cubic grid box (30 Å × 30 Å × 30 Å) was centered on the active sites of SIRT1 and PGC-1α to accommodate ligand flexibility, with grid spacing set to 0.05 nm for precise spatial sampling. Blind docking simulations were executed using AutoDock Vina 1.2.2 (http://autodock.scripps.edu/) under default parameters. The lowest-energy poses were subsequently selected for visualization and interaction analysis.

### 2.9 Surface plasmon resonance (SPR) analysis

Analysis of direct interactions between COS and SIRT1, or PGC-1α was performed at 25 °C on a BIAcore S2100 SPR instrument (GE Healthcare). SPR running buffer contained PBS (pH 7.4) and was prepared immediately before measurement. SIRT1 or PGC-1α was immobilized via amine coupling on a flow cell of the chip. The remaining binding sites on the chips were blocked by 1 mol L^−1^ ethanolamine (pH 8.5) at a flow rate of 10 Ul min^−1^ for 7 min. Control sensor-grams, obtained on an empty flow cell where the coupling reaction had been conducted in the presence of coupling buffer alone, were always subtracted from binding responses. COS was diluted in the running buffer and then injected at different concentrations and passed over adjacent target and control flow cells at a flow rate of 30 μL min^−1^ for 180 s, and the decomposition period was adjusted to 300 s. The bound analytes were removed by a 30-s wash with running buffer.

### 2.10 WOC-mediated mitochondrial transfer on angiogenesis

Six experimental groups based on Transwell system were established: untreated HUVECs (Control), H_2_O_2_-injured HUVECs (HUVECs + H_2_O_2_), macrophage-exposed HUVECs (Mφ@HUVECs), WOC-primed macrophages with HUVECs [(Mφ+WOC)@HUVECs], WOC-primed macrophages with injured HUVECs [(Mφ+WOC)@(HUVECs + H_2_O_2_)], and Dynasore (100 μM)-inhibited counterparts [(Mφ+WOC + Dynasore)@(HUVECs + H_2_O_2_)]. HUVECs viability was assessed via CCK-8 assay (days 1, 3, 5), while angiogenesis capacity was evaluated through cell wound scratch assay (250 μm width) and Matrigel tube formation assays (6 h imaging, quantified via ImageJ). Mitochondrial transfer was confirmed by labeling macrophages with MitoTracker Deep Red FM, followed by flow cytometry and fluorescence microscopy to detect mitochondrial uptake in HUVECs. TEM was used to further confirm vesicles-dependent mitochondrial transfer. Further HUVECs mitochondrial function analyses included ROS detection via DCFH-DA probe, MMP assessment using JC-1 fluorescence, and measurement of SOD/CAT activities according to the instructions. qRT-PCR was used to analyze angiogenic gene expression (SIRT1, PGC-1α, and VEGF). Autophagy-related proteins Pink1/Parkin in macrophages was detected via fluorescence microscopy and Western blotting.

### 2.11 In vivo wound healing assay

All animal procedures were conducted in accordance with protocols approved by the Animal Ethics Committee of Guangzhou University of Chinese Medicine (Approval No. SYXK 2021-0059) and adhered to the ARRIVE guidelines. Type 1 diabetes was induced in male Sprague-Dawley rats (8 weeks, 200–220 g) via intraperitoneal injection of streptozotocin (60 mg kg^−1^, dissolved in 0.05 M sodium citrate buffer, pH 4.5) administered daily for 5 consecutive days after a 12-h fast. Blood glucose levels were monitored weekly using a glucometer, with diabetes confirmed by sustained hyperglycemia (>16.7 mmol L^−1^) for 30 days.

Diabetic rats were randomized into four treatment groups: Control (PBS), COS (dosage: 50 mg kg^−1^), GSH (dosage: 100 mg kg^−1^), and WOC (dosage: 100 mg kg^−1^). Treatments were sprayed topically once on day 0 post-wounding. Under aseptic conditions, rats were anesthetized with 3 % sodium pentobarbital (1 mL kg^−1^, i.p.), and dorsal hair was removed followed by disinfection with 75 % ethanol. Four full-thickness excisional wounds (10 mm diameter) were created using a sterile biopsy punch. Wound progression was documented via standardized digital photography (Canon EOS 90D) at days 0, 3, 7, and 14. Wound areas were quantified using ImageJ, with healing rates calculated as:

$$\text{Wound}\;\text{healing}\;\text{rate}\;\left(\%\right)=\frac{A_{0}-A_{t}}{A_{0}}\times 100\%$$

where *A*_*0*_ and *A*_*t*_ represent initial and time-dependent wound areas, respectively. At endpoint, peri-wound tissues were harvested for histopathological (H&E, Masson's trichrome) and molecular analyses.

### 2.12 In vivo histopathological analysis

Full-thickness wound tissues were harvested at days 3, 7, and 14, fixed in 4 % paraformaldehyde (24 h, 4 °C), dehydrated through an ethanol-xylene gradient, and paraffin-embedded. Sections were stained with hematoxylin and eosin (H&E) for re-epithelialization assessment and Masson's trichrome for collagen fiber analysis. Immunohistochemical (IHC) staining was performed for HIF-1α, VEGF, CD31, F4/80, CD206, IL-10, TNF-α, SIRT1, and PGC-1 using antigen retrieval (pH 6.0 citrate buffer, 95 °C, 20 min) and DAB visualization (Leica Bond RX). The scar elevation index (SEI) was employed to assess the efficacy of WOC in inhibiting scar formation:

$$\text{SEI}=\frac{Total\;Wound\;Dermis\;\left(TWD\right)\;}{Normal\;Dermis\;\left(ND\right)}$$

Where *TWD* represents the dermal height at the healing wound, while the *ND* refers to the dermal height of normal tissue adjacent to the wound. SEI = 1 indicates a non-scarring wound repair, whereas SEI >1 indicates hypertrophic scar on the wound.

### 2.13 Statistical analysis

Data are shown as means ± SD. Differences between two groups were analyzed by Student's *t*-test. One-way analysis of variance (ANOVA) was used to assess the difference between multiple groups. P < 0.05 was considered statistically significant.

## 3 Results and discussion

### 3.1 Synthesis, morphology, and stimuli-responsive characterization

**Fig. 1:**
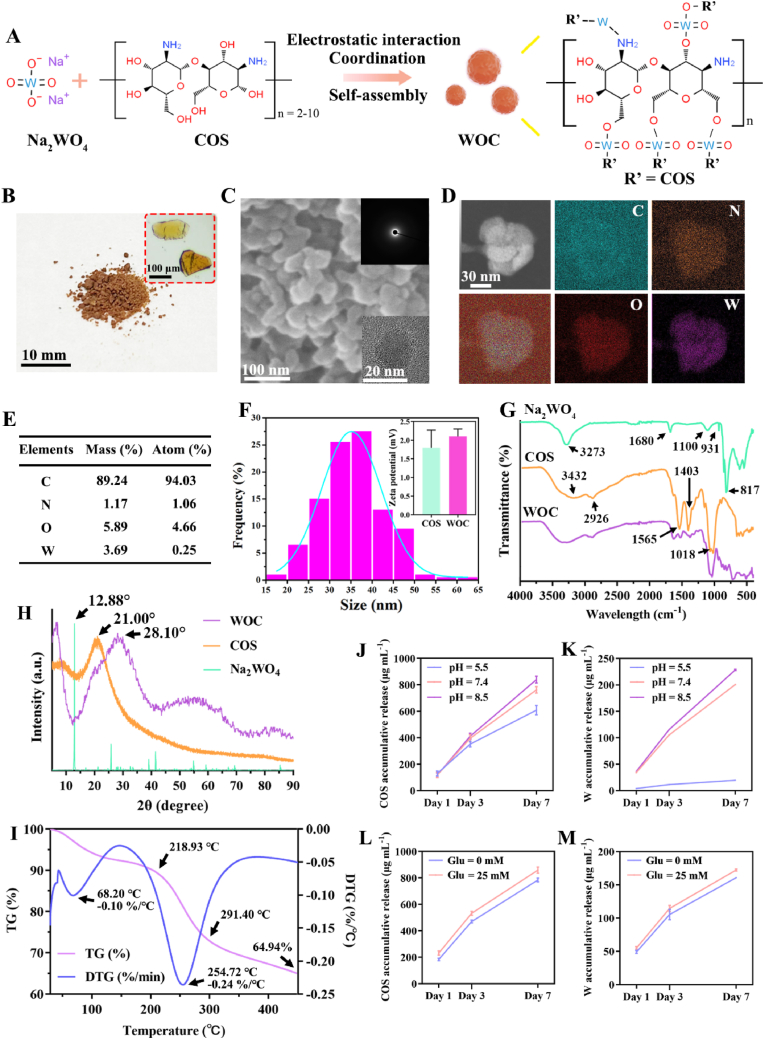
Synthesis and characterization of WOC nanoplatform. (A) Schematic illustration of the one-pot coordination-driven assembly process for WOC synthesis; (B) Macroscopic appearance of WOC showing characteristic brown coloration in its aggregated state. (C) SEM/TEM images revealing spherical morphology with amorphous structure confirmed by SAED (inset); (D–E) EDS elemental mapping demonstrating homogeneous distribution of W, C, N, and O; (F) Hydrodynamic size distribution and Zeta potential profiles. (G) FTIR spectra identifying key vibrational modes; (H) XRD patterns showing loss of Na_2_WO_4_ crystallinity and emergence of amorphous WOC halo; (I) Thermal stability profile with three-stage decomposition; (J–K) pH-dependent WO_4_^2−^/COS release (n = 12); (L–M) Glucose-accelerated release due to competitive displacement (n = 12). (For interpretation of the references to color in this figure legend, the reader is referred to the Web version of this article.)

The WOC nanoplatform was synthesized via a one-pot coordination-driven assembly of Na_2_WO_4_ and COS, involving electrostatic interaction, Lewis acid-base interactions, and self-assembly (Fig. 1A). The Na_2_WO_4_ exhibits weak basicity, and its hydrolysis in aqueous solution leads to the formation of OH^−^ ions, which can induce physical cross-linking of COS. Additionally, the consumption of OH^−^ ions promotes the forward reaction of WO_4_^2−^ hydrolysis, producing more H_2_WO_4_ molecules. On the other hand, the tungstate group acts as a Lewis acid, strongly interacting with -NH_2_ groups (Lewis bases, possessing lone electron pairs) [33]. This enables rapid coordination reactions between WO_4_^2−^ and -NH_2_ of COS [34], promoting covalent interactions between -OH and WO_4_^2−^.

In the aggregated state, WOC exhibited a brownish appearance (Fig. 1B). SEM/TEM revealed monodisperse spherical nanoparticles with an average diameter of 35.56 ± 7.40 nm (Fig. 1C–D). Selected-area electron diffraction (SAED) confirmed the amorphous nature of WOC, as evidenced by the absence of crystalline lattice fringes (Fig. 1C, **inset**). EDS elemental mapping confirmed homogeneous distribution of C (89.24 wt%), N (1.17 wt%), O (5.89 wt%), and W (3.69 wt%), validating successful integration of WO_4_^2−^ and COS (Fig. 1D–E). DLS showed a hydrodynamic size range of 20–50 nm and an average zeta potential of +2.10 mV (Fig. 1F), consistent with the cationic COS backbone.

As shown in Fig. 1G, the FTIR spectrum revealed characteristic absorption peaks of COS at the wavelengths of 3432 cm^−1^ and 2926 cm^−1^, which can be attributed to the O-H (overlapped with N-H asymmetrical stretch) and aliphatic C-H asymmetric stretching vibrations [35]. Additionally, the peaks observed at 1565 cm^−1^, 1403 cm^−1^ and 1018 cm^−1^ correspond to the N-H bending of amines, -OH plane bending of alcohols, C-O-C bonds, C-N stretching vibration and N-H oscillating respectively [36]. The characteristic peaks of these N-H and O-H bending in WOC became wider and weaker, which proved that -OH and -NH_2_ in COS molecules reacted with WO_4_^2−^. Emergence of a W-O-W stretching vibration at 817 cm^−1^ (absent in COS/and Na_2_WO_4_ controls) confirmed tungstate polymerization [37].

The XRD analysis (Fig. 1H) revealed the disappearance of Na_2_WO_4_'s crystalline peak at 12.88° (2θ) and COS's broad peak at 21°, confirming structural reorganization [38]. A new amorphous halo at 2θ = 50°–60° was attributed to the altered crystal structure due to the reaction between WO_4_^2−^ and -NH_2_/-OH groups in COS [38]. TGA demonstrated three-stage decomposition: hydration loss, polymer degradation, and inorganic residue (Fig. 1I). The weight loss below 100 °C is due to hydration loss, accounting for 7–8 %. A second weight loss from 218.93 °C to 291.40 °C, about 25 % of the total, is caused by WOC decomposition. DTG curves show double peaks at 68.20 °C and 254.72 °C. At 450 °C, the residual mass of WOC is approximately 64.94 %.

The pathogenesis of DW creates a complex microenvironment with high glucose, insufficient oxygen, inflammation, as well as pH variations (7.0–8.9) [[39], [40], [41]]. As depicted in Fig. 1J and K, COS and W release increased at pH 7.4 and 8.5, with higher release at pH 8.5. The release mechanism involves tungstate hydrolysis. Alkaline conditions enhance hydrolysis via ionic bases, which shift the equilibrium and accelerate kinetics. Increased OH- ions further promote tungstate hydrolysis. In contrast, neutral or acidic conditions slow the reaction due to the lack of ionic bases. Notably, glucose enhances COS and W release compared to PBS (pH 7.4), likely due to competitive interactions between COS and glucose (Fig. 1L–M).

### 3.2 Antioxidant activity

**Fig. 2:**
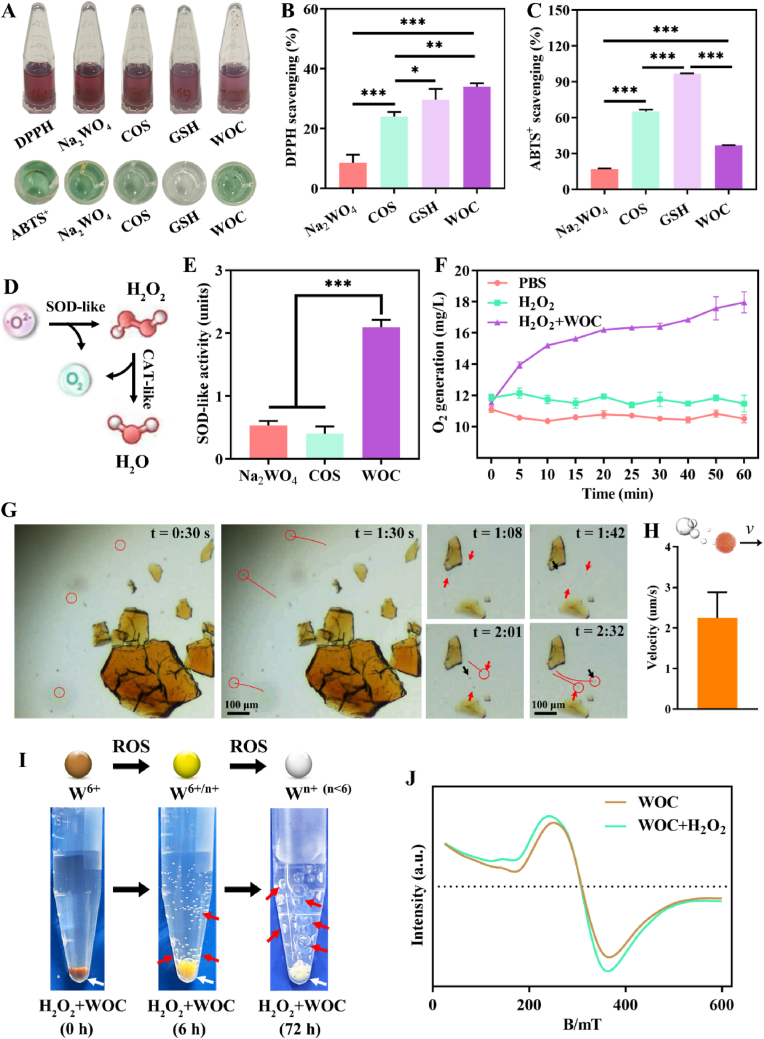
Antioxidant and nanozyme activities of WOC nanoplatform. (A) Schematic of radical scavenging assays using DPPH and ABTS^+^; (B) Quantitative DPPH and (C) ABTS^+^ scavenging activity of WOC (n = 3); (D) Catalytic mechanisms of SOD-like (O_2_^−^ disproportionation) and CAT-like (H_2_O_2_ decomposition) activities; (E) SOD like activity (n = 3); (F) CAT-mimetic kinetics showing time-dependent H_2_O_2_ degradation (n = 3); (G) Optical microscopy visualization of WOC's non-Brownian locomotion driven by oxygen microbubble propulsion; (H) Quantitative velocity analysis confirming directional movement (n = 6); (I) Real-time colorimetric response to H_2_O_2_ correlating with W valence transitions; (J) EPR spectra verifying ROS-responsive electron transfer. Data represent mean ± SD; Significance: ∗p < 0.05, ∗∗p < 0.01, ∗∗∗p < 0.001.

The antioxidant capacity of WOC was evaluated via free radical scavenging assays using DPPH and ABTS^+^, with GSH included as a positive control due to its established ROS-scavenging efficacy (Fig. 2A) [42]. Quantitative analysis revealed that WOC outperformed both its individual components and GSH in DPPH scavenging, achieving a radical elimination rate of 33.98 ± 0.91 % at 2 mg/mL, compared to 29.61 ± 2.93 % for GSH and 24.03 ± 1.23 % for COS (Fig. 2B). In ABTS^+^ assays, WOC demonstrated superior activity relative to Na_2_WO_4_ controls (scavenging rate: 36.60 ± 0.37 % vs. 16.88 ± 0.61 %), though lower than COS (65.09 ± 1.38 %) and GSH (96.60 ± 0.01 %), confirming its robust antioxidant potential (Fig. 2C).

WOC further exhibited dual enzyme-mimetic activity critical for ROS regulation: SOD-like behavior catalyzing O^2−^ disproportionation into O_2_ and H_2_O_2_, and CAT-like activity decomposing H_2_O_2_ into H_2_O and O_2_ (Fig. 2D). The SOD-like activity of WOC was found to be remarkably high (2.1 ± 0.10 units), surpassing both its individual components (Fig. 2E). CAT-like functionality was evidenced by time-dependent O_2_ generation using dissolved oxygen meter (Fig. 2F). The catalytic decomposition of H_2_O_2_ by WOC generates oxygen microbubbles, which impart directed kinetic energy to the WOC particles. Under optical microscopy, this propulsive force induces non-Brownian locomotion of WOC particles, characterized by linear trajectories and arc-shaped motility at an average velocity of 2.25 ± 0.58 μm/s (Fig. 2G–H; Supporting media). This directed motion confirms enzyme-like chemomechanical energy conversion.

A concomitant visual transition in WOC's colorimetric profile (Fig. 2I, Fig. S1) correlated with H_2_O_2_ concentration and scavenging but pH value, attributed to valence-state decrease of W as confirmed by EPR spectroscopy (g-factor = 2.183, 200–400 mT; Fig. 2J).

### 3.3 Macrophage polarization and mitochondrial modulation

**Fig. 3:**
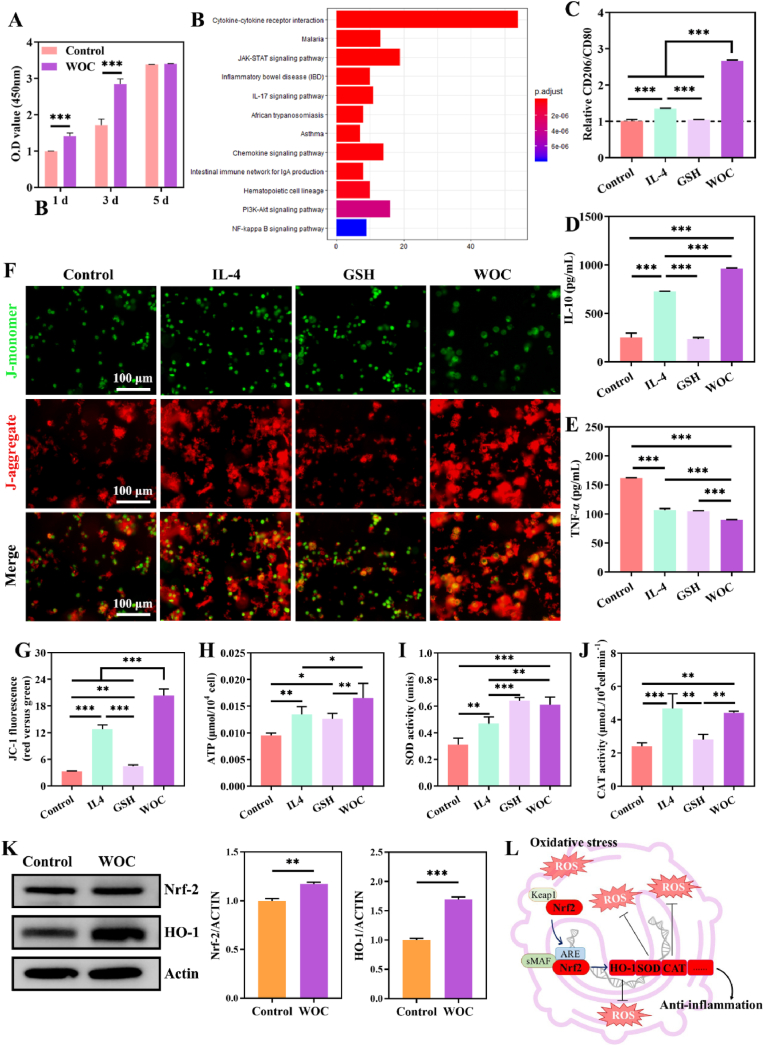
WOC enhances macrophage mitochondrial function to drive anti-inflammatory polarization. (A) WOC enhanced RAW264.7 viability and proliferation (n = 4); (B) KEGG pathway enrichment of macrophage supernatants identified upregulated anti-inflammatory signaling; (C) Flow cytometry revealed a significant increase in CD206/CD80 ratio (n = 4); (D–E) Cytokine profiling showed elevated IL-10 and reduced TNF-α (n = 4); (F–G) JC-1 staining demonstrated higher MMP via JC-1 red/green fluorescence ratio (n = 4); (H) ATP production (n = 4); (I) SOD activity (n = 4) and (J) CAT activity (n = 3); (K–L) Nrf-2/HO-1 pathway activation mediated redox homeostasis and anti-inflammatory responses (n = 3). Data represent mean ± SD; Significance: ∗p < 0.05, ∗∗p < 0.01, ∗∗∗p < 0.001. (For interpretation of the references to color in this figure legend, the reader is referred to the Web version of this article.)

IL-4-treated macrophages were employed as M2-polarized positive controls. The cytotoxicity of WOC was assessed using the CCK8 test. Cytotoxicity assessment via CCK-8 assay confirmed WOC's biosafety, with RAW264.7 cells exhibiting proliferation stimulation on day 3 (Fig. 3A). Proteomic profiling of WOC-treated macrophage supernatants revealed differential protein expression (DEPs) significantly enriched in anti-inflammatory pathways (e.g. JAK-STAT, IL-17, PI3K-AKT, and NF-κB) through KEGG analysis (Fig. 3B). Flow cytometry demonstrated a 2.67-fold increase in the CD206^+^/CD80^+^ ratio (p < 0.001) for WOC-treated macrophages versus controls (Fig. 3C, Fig. S3), confirming M2 polarization driven by WOC. WOC induced a potent immunomodulatory response, elevating IL-10 secretion to 961.67 ± 5.44 pg mL^−1^ (vs. 242.5 ± 34.99 pg mL^−1^ control) while suppressing TNF-α to 89.73 ± 0.47 pg mL^−1^ (vs. 161.73 ± 0.54 pg mL^−1^ control) over 72 h, outperforming IL-4 and GSH treatments (Fig. 3D–E).

Mitochondrial OXPHOS serves as a central regulator of macrophage polarization and inflammatory response dynamics [15]. To delineate WOC's anti-inflammatory mechanism, we evaluated its impact on mitochondrial bioenergetics in RAW264.7 macrophages co-cultured with WOC for 72 h. Quantitative profiling revealed significant enhancements in MMP, ATP synthesis, and antioxidant enzyme activities. JC-1 staining, which exhibits potential-dependent J-aggregate formation, demonstrated a 6.23-fold increase in red/green fluorescence ratio (20.32 ± 1.30 vs. 3.26 ± 0.09 control; Fig. 3F–G), confirming MMP polarization that augments the proton gradient for ATP generation (Fig. 3H). This bioenergetic shift correlated with elevated ATP levels, underscoring WOC's capacity to amplify OXPHOS-driven energy production.

The antioxidant enzymes SOD and CAT, which is critical for scavenging O_2_^−^ and H_2_O_2_ [43], were synergistically upregulated by WOC. SOD catalyzes the dismutation of O_2_^−^ into O_2_ and H_2_O_2_, whereas CAT facilitates the decomposition of H_2_O_2_ into O_2_ and H_2_O. Collectively, these enzymes safeguard mitochondria against oxidative stress. SOD activity surged by 1.95-fold versus controls (p < 0.001), while CAT activity increased 1.84-fold (p < 0.01), effectively mitigating mitochondrial oxidative stress (Fig. 3I–J). Furthermore, WOC activated the Nrf-2/HO-1 pathway, upregulating Nrf-2 expression by 1.17-fold and HO-1 by 1.69-fold (Fig. 3K–L), which are master regulators of cellular antioxidant defenses and anti-inflammatory responses. These coordinated enhancements in redox homeostasis conclusively demonstrate WOC's dual role in driving M2 polarization through metabolic reprogramming and oxidative stress resolution.

**Fig. 4:**
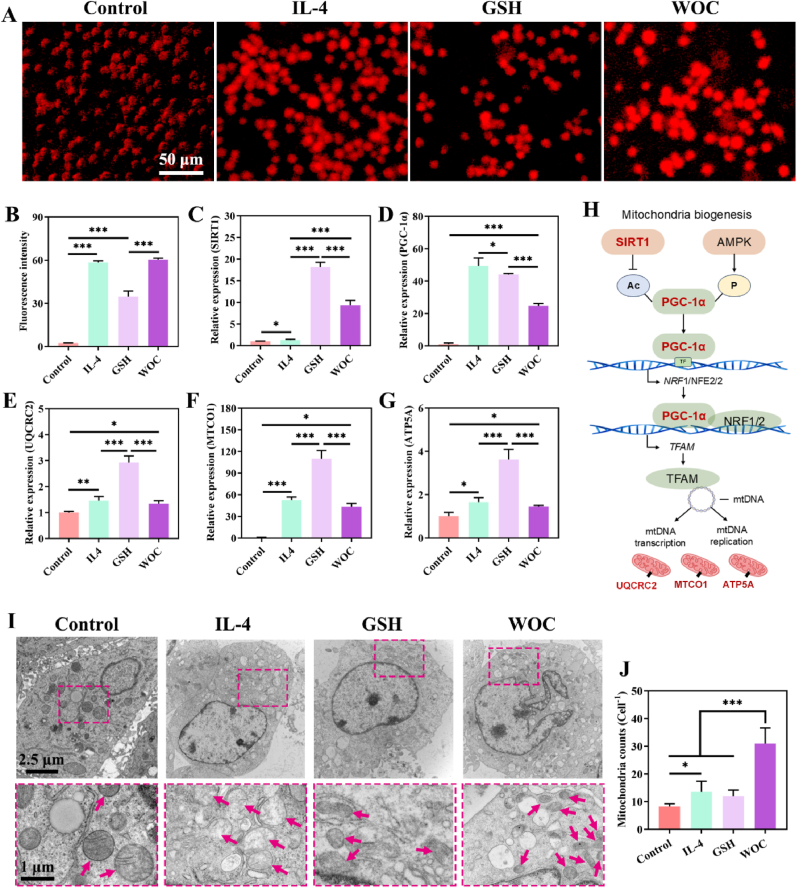
WOC enhances mitochondrial biogenesis. (A–B) Mitochondrial mass quantification via MitoTracker Deep Red FM fluorescence (n = 4); (C) qRT-PCR profiling of mitochondrial biogenesis markers (n = 3): (C) SIRT1, (D) PGC-1α; (E) UQCRC2; (F) MTCO1, and (G) ATP5A; (H) Schematic of SIRT1/PGC-1α axis driving mitochondrial biogenesis; (I) TEM images of macrophages and mitochondrial ultrastructure (red arrows), and (J) Mitochondrial counts from TEM images (n = 4). Data represent mean ± SD; Significance: ∗p < 0.05, ∗∗p < 0.01, ∗∗∗p < 0.001. (For interpretation of the references to color in this figure legend, the reader is referred to the Web version of this article.)

Mitochondrial mass in macrophages was quantified using MitoTracker Deep Red FM probe, revealing a more than 20-fold increase in fluorescence intensity in WOC-treated and IL-4 groups compared to controls (p < 0.001; Fig. 4A–B), confirming WOC's capacity to amplify mitochondrial biogenesis and functionality. Central to this process is the SIRT1/PGC-1α axis: SIRT1, a NAD^+^-dependent deacetylase regulating mitochondrial biogenesis, synergizes with PGC-1α—a master transcriptional coactivator of oxidative metabolism—to enhance mitochondrial respiratory capacity and oxidative stress resilience [44,45]. SIRT1/PGC-1 axis critically governs macrophage polarization by coupling metabolic reprogramming to anti-inflammatory signaling (Fig. 4H).

Mitochondrial OXPHOS relies on five inner membrane-bound complexes (I, II, III_2_, IV, and V), where complexes I, II, III_2_, and IV mediate electron transport, and complex V (ATP synthase) harnesses the proton gradient for ATP synthesis. The subunits UQCRC2, MTCO1 and ATP5A belong to mitochondrial complexes III_2_, IV and V, respectively [46]. qRT-PCR analysis demonstrated WOC's profound upregulation of SIRT1, PGC-1α, UQCRC2, MTCO1, and ATP5A compared to the Control group (Fig. 4C–G). Biological TEM further corroborated these findings, demonstrating a 3-fold increase in mitochondrial density (red arrows) within WOC-treated macrophages (31 ± 4.55 mitochondria/cell vs. 8 ± 0.83 in controls; p < 0.001), with ultrastructural preservation of cristae architecture and matrix integrity (Fig. 4I–J). These coordinated enhancements in mitochondrial biogenesis correlate with amplified OXPHOS activity and underscore WOC's unique ability to rewire macrophage metabolism. By augmenting SIRT1/PGC-1α-driven mitochondrial biogenesis and respiratory chain efficiency, WOC establishes a robust anti-inflammatory microenvironment critical for resolving the inflammatory phase of diabetic wound healing.

**Fig. 5:**
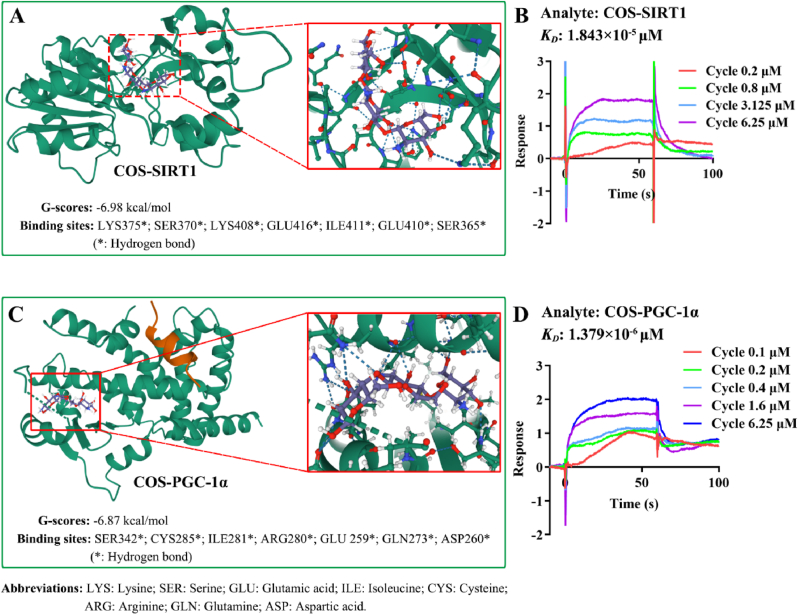
Molecular docking analysis and SPR between COS and SIRT1, or PGC-1α. (A) High-affinity binding of COS to SIRT1 catalytic domain, exhibiting a binding energy of −6.98 kcal mol^−1^. Key interactions include hydrogen bonds with LYS375, SER370, LYS408, GLU416, ILE411, GLU410, and SER365; (B) SPR revealed the corresponding *K*_*D*_ between COS and SIRT1 were 1.843 × 10^−5^ μM. (C) Stable binding of COS to PGC-1α′s transcriptional activation domain (−6.87 kcal mol^−1^), mediated by hydrogen bonding with SER342, CYS285, ILE281, ARG280, GLU259, GLN273, and ASP260; (D) SPR revealed the corresponding *K*_*D*_ between COS and PGC-1α were 1.379 × 10^−6^ μM. These interactions mechanistically support WOC's observed upregulation of SIRT1 and PGC-1α in Fig. 4, positioning COS as an allosteric modulator of mitochondrial biogenesis.

The transcription factors, SIRT1 and PGC-1α, regulate genes involved in various cellular processes including the respiratory chain, mitochondrial transcription, translation and replication machinery, as well as protein import and assembly apparatus [47]. To elucidate the molecular basis of WOC's bioactivity, we performed molecular docking (Autodock Vina v.1.2.2) and SPR to evaluate COS (the bioactive component of WOC) for its binding affinity to SIRT1 and PGC-1α (Fig. 5A and C). Structural analysis revealed that COS formed visible hydrogen bonds and strong electrostatic interactions with its protein targets. Additionally, the hydrophobic pockets of each target were effectively occupied by COS. Notably, COS@SIRT1 exhibited a low binding energy of −6.98 kcal/mol, while COS@PGC-1α demonstrated a similarly stable binding with a low binding energy of −6.87 kcal mol^−1^ [48]. The low binding energies (G-score < −6.5 kcal mol^−1^) indicate robust molecular recognition. Further, the SPR *K*_*D*_ value of the interaction between COS and SIRT1, or PGC-1α has been calculated at 1.843 × 10^−5^ μM and 1.379 × 10^−5^ μM, respectively (Fig. 5B and D). This shows that COS exhibit high affinity toward the target SIRT1, or PGC-1α. The strength of this specific interaction is comparable to natural recognition components such as antibodies [49]. These interactions position COS to allosterically modulate SIRT1/PGC-1α activity, mechanistically linking WOC's mitochondrial enhancements to transcriptional regulation of oxidative metabolism.

### 3.4 Angiogenic activity of mitochondria transferred from WOC-polarized macrophage

**Fig. 6:**
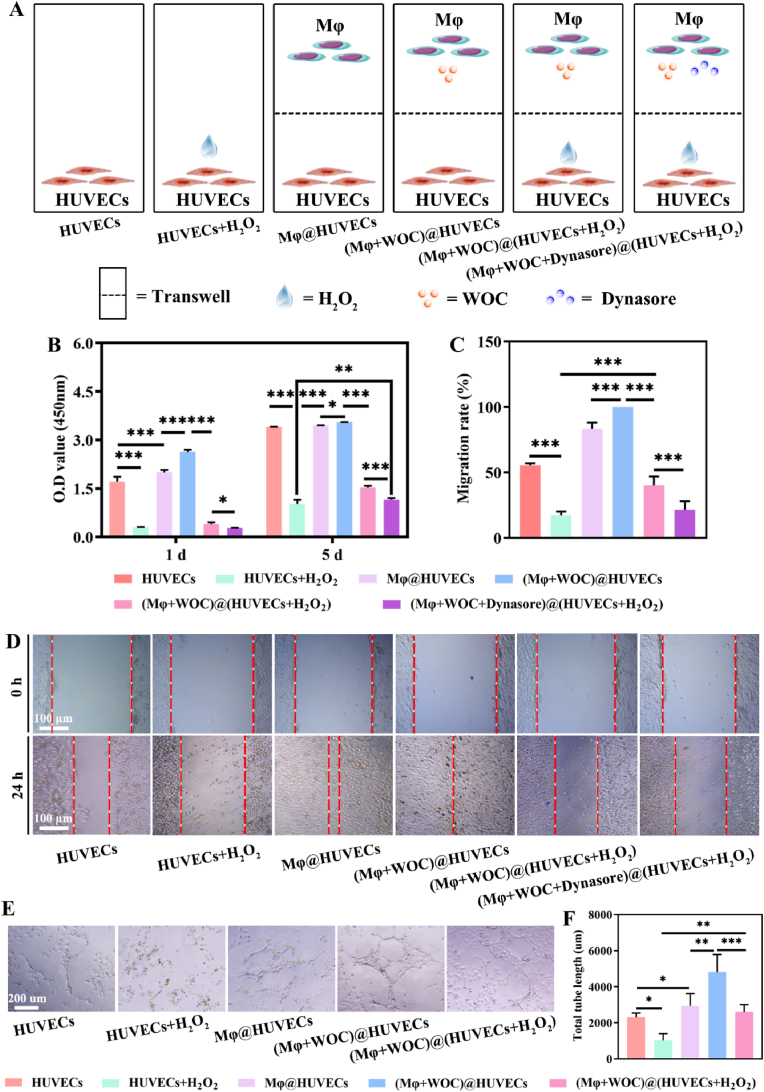
WOC-polarized M2 macrophages rescue angiogenic ability of H_2_O_2_-injured HUVECs. (A) Schematic representation of co-culture grouping of macrophages and endothelial cells; (B) Cell viability of HUVECs (n = 4); (C–D) Cell migration of HUVECs (n = 4). (E–F) Tube formation of HUVECs. Data presented as mean ± SD. Significance: ∗P < 0.05, ∗∗P < 0.01, ∗∗∗P < 0.001.

Revascularization via functional angiogenesis is critical for DW healing, particularly under oxidative stress. To investigate mitochondrial transfer-mediated endothelial repair, we employed a Transwell co-culture system pairing RAW264.7 macrophages with HUVECs, the latter subjected to H_2_O_2_-induced injury (440 mM, 1 h). Dynasore, a non-competitive inhibitor of dynamin 1/2 and mitochondrial dynamin GTPases that blocks vesicle scission and mitochondrial transfer, served to isolate transfer-dependent effects [50]. Experimental groups included: untreated HUVECs (Control), H_2_O_2_-injured HUVECs (HUVECs + H_2_O_2_), macrophage-exposed HUVECs (Mφ@HUVECs), WOC-primed macrophages with HUVECs [(Mφ+WOC)@HUVECs], WOC-primed macrophages with injured HUVECs [(Mφ+WOC)@(HUVECs + H_2_O_2_)], and Dynasore-inhibited counterparts [(Mφ+WOC + Dynasore)@(HUVECs + H_2_O_2_)] (Fig. 6A). WOC-polarized M2 macrophages significantly enhanced H_2_O_2_-injured HUVECs viability (1.5-fold increase) versus untreated HUVECs (1.04-fold increase) at day 5, which Dynasore inhibition abrogated 25 % viability (p < 0.001) (Fig. 6B). Migration assays mirrored these trends, with injured HUVECs co-cultured with WOC-primed macrophages achieving 40.29 ± 5.85 % wound closure at 24 h versus 17.33 ± 2.35 % in injured controls (p < 0.001). Dynasore inhibition attenuated this pro-migratory effect by 46.3 % (p < 0.001) (Fig. 6C–D). Tube formation assays further validated WOC's angiogenic rescue: injured HUVECs co-cultured with WOC-treated macrophages developed 2594.40 ± 351.78 μm of tube formation at 6 h, surpassing H_2_O_2_ injured controls (1025.67 ± 303.41 μm, p < 0.01) by 2.5-fold (Fig. 6E–F). These findings suggest that WOC-polarized M2 macrophages can rescue angiogenic ability of H_2_O_2_-injured HUVECs.

**Fig. 7:**
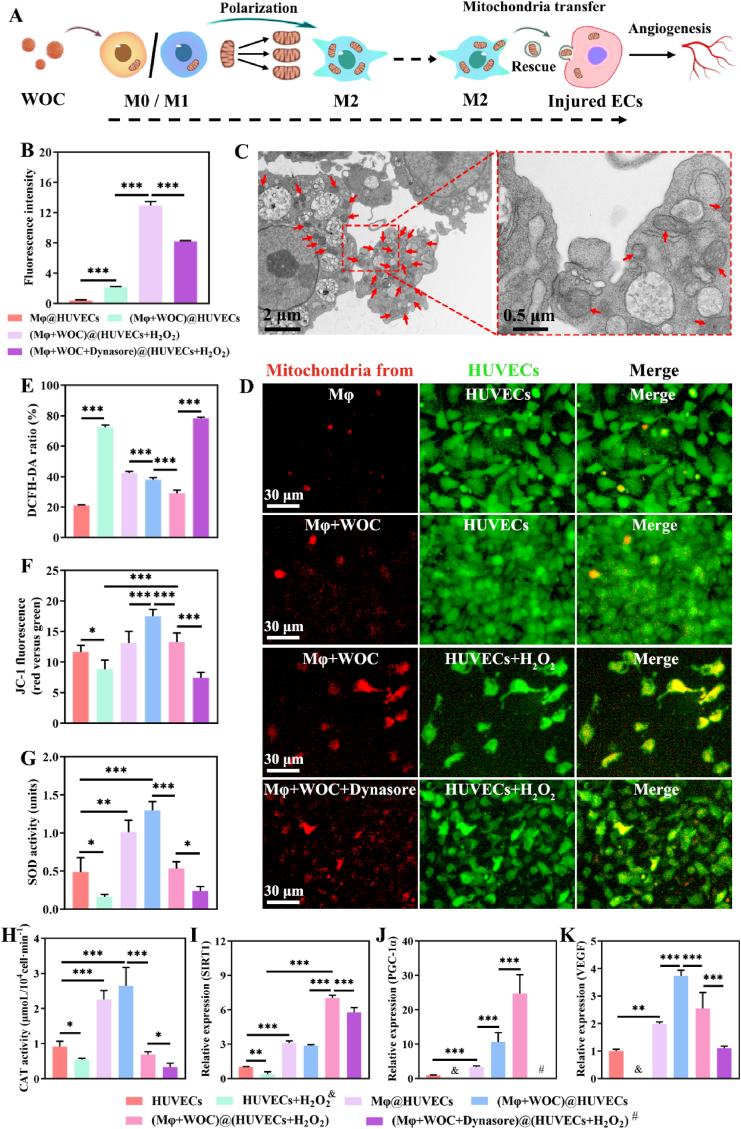
Mitochondrial transfer from WOC-polarized M2 macrophages promotes angiogenesis in HUVECs. (A) Schematic illustrating the therapeutic role of mitochondria derived from WOC-primed M2 macrophages in enhancing EC angiogenesis. (B) Mitochondrial fluorescence intensity in HUVECs following uptake of macrophages' mitochondria, quantified via flow cytometry (n = 3); (C) SEM image showing mitochondria (red arrows) encapsulated within microvesicles secreted by M2 macrophages; (D) Mitochondrial fluorescence images of HUVECs after endocytosed the labeled mitochondria from macrophage; (E) Total ROS levels in HUVECs measured by DCFH-DA assay (n = 5). (F) MMP in HUVECs assessed via JC-1 red/green fluorescence ratio (n = 5). (G) SOD and (H) CAT activity in HUVECs under indicated treatments (n = 3). (I–K) qRT-PCR analysis of SIRT1, PGC-1α, and VEGF gene expression in HUVECs (n = 3); The symbol “&” “#” indicates undetectable expression levels. Data presented as mean ± SD. Significance: ∗P < 0.05, ∗∗P < 0.01, ∗∗∗P < 0.001. (For interpretation of the references to color in this figure legend, the reader is referred to the Web version of this article.)

As described, WOC significantly boosted mitochondrial OXPHOS in macrophages, promoting M2 polarization and restoring angiogenic ability of H_2_O_2_-injured HUVECs. This finding leads us to hypothesize that mitochondrial transfer from WOC-primed M2 macrophages mediates endothelial repair (Fig. 7A). To validate this, Mitotracker Deep Red FM-labeled macrophages were co-cultured with HUVECs, revealing a 6.06-fold increase in mitochondrial fluorescence intensity in H_2_O_2_-injured HUVECs [(Mφ+WOC)@(HUVECs + H_2_O_2_)] versus non-injured controls [(Mφ+WOC)@HUVECs] via flow cytometry (Fig. 7B). The TEM further visualized mitochondria encapsulated within extracellular microvesicles shed by WOC-stimulated macrophages (Fig. 7C), suggesting vesicle-mediated mitochondrial transfer. Furthermore, spatial analysis revealed that HUVECs exhibit mitochondrial tropism, demonstrating a higher cellular density within mitochondrial-enriched regions compared to mitochondrial-depleted zones (Fig. S2). Strikingly, Dynasore pretreatment attenuated mitochondrial fluorescence intensity by 36.5 % (p < 0.001) in injured HUVECs (Fig. 7D), confirming dynamin-dependent transfer mechanisms.

Functional profiling of HUVECs co-cultured with WOC-primed M2 macrophages revealed a multi-modal rescue of mitochondrial homeostasis under oxidative stress. ROS quantification via DCFH-DA demonstrated a 59.9 % reduction in H_2_O_2_-injured HUVECs in the presence of WOC-primed M2 macrophages (Mφ+WOC) versus untreated injury controls (HUVECs + H_2_O_2_) (p < 0.001), outperforming all groups except baseline controls (Fig. 7E). JC-1 fluorescence ratios (red/green) served as a quantitative measure of MMP, where higher ratios indicate greater mitochondrial polarization. Compared to control groups (Control, HUVECs + H_2_O_2_, and Mφ@HUVECs), HUVECs co-cultured with WOC-primed macrophages (Mφ+WOC@HUVECs) demonstrated a significant increase in MMP (p < 0.001). Importantly, this mitochondrial rescue effect was particularly evident in injured HUVECs, where the (Mφ+WOC)@(HUVECs + H_2_O_2_) group maintained 113.8 % of baseline MMP - a 1.78-fold improvement over Dynasore-treated counterparts (Mφ+WOC + Dynasore)@(HUVECs + H_2_O_2_) (p < 0.001). Antioxidant defenses (SOD and CAT activity) were similarly enhanced as depicted in Fig. 7G–H. These findings provide further confirmation that mitochondria transferred from WOC-stimulated macrophages can enhance OXPHOS and antioxidant capacity in injured HUVECs.

SIRT1 and PGC-1α are recognized as positive regulators of mitochondrial biogenesis and function, while vascular endothelial growth factor (VEGF) serves as a potent stimulator of angiogenesis. The expression levels of PGC-1α and VEGF in HUVECs were severely impaired by H_2_O_2_ (440 mM, 1 h). Conversely, SIRT1, PGC-1α, and VEGF were upregulated in the presence of WOC-primed M2 macrophages (Mφ+WOC). Dynasore-mediated inhibition markedly attenuated these effects, directly linking mitochondrial transfer to transcriptional activation of angiogenic and bioenergetic programs (Fig. 7I–K). These findings establish WOC's capacity to coordinate intercellular mitochondrial transfer, coupling metabolic reprogramming with redox homeostasis to restore endothelial functionality.

**Fig. 8:**
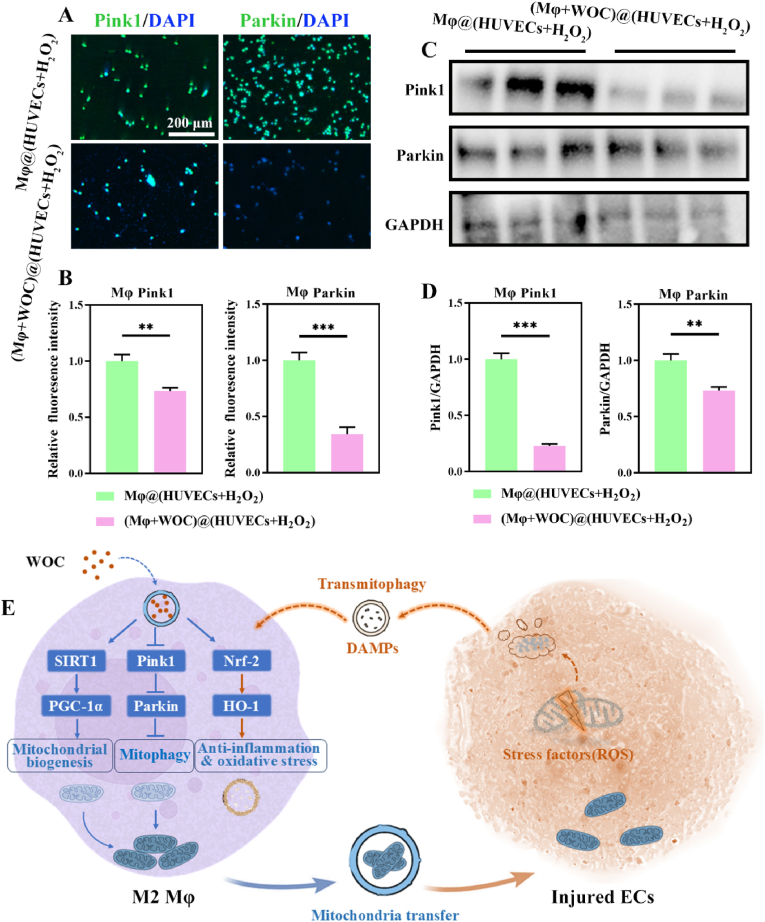
Proposed mechanisms of WOC-mediated mitochondrial transfer. (A–B) Representative immunofluorescence images and quantitative analysis of Pink1/Parkin expression (n = 3). (C–D) WB bands and densitometric quantification of Pink1/Parkin levels (n = 3). (E) Schematic model depicting the "increase income and reduce expenditure" mechanism underlying WOC-mediated mitochondrial transfer. Data presented as mean ± SD. Significance: ∗P < 0.05, ∗∗P < 0.01, ∗∗∗P < 0.001. (For interpretation of the references to color in this figure legend, the reader is referred to the Web version of this article.)

The results above indicate that injured endothelial cells enhance mitochondrial transfer capacity in WOC-primed M2 macrophages. Due to mitochondria's bacterial ancestry, they and their components can be recognized as damage-associated molecular patterns (DAMPs), such as depolarized mitochondria, mitochondrial formyl peptide, and mtDNA, by the immune cells, leading to inflammation. Recipient cells activate Pink1/Parkin axis mediated-mitophagy to eliminate these proinflammatory DAMPs upon their uptake [51,52]. As demonstrated in Fig. 8A–D, macrophages exposed to H_2_O_2_ injured-HUVECs in the Transwell system exhibited upregulated expression of the Pink1/Parkin axis. However, WOC-primed macrophages displayed significant attenuation of Pink1/Parkin expression. Integrating these observations with enhanced Nrf2/HO-1 antioxidative signaling (Fig. 3K–L) and elevated mitochondrial biogenesis in macrophages (Fig. 4), we propose that WOC orchestrates mitochondrial transfer via a dual "increase income and reduce expenditure" mechanism: (1) It stimulates mitochondrial biosynthesis through SIRT1/PGC-1α axis activation, while (2) suppressing mitophagy via Nrf2/HO-1-mediated DAMPs scavenging, thereby downregulating Pink1/Parkin. This coordinated regulation ultimately enhances intercellular mitochondrial transfer, as depicted in Fig. 8E.

### 3.5 Assessment of DW healing efficacy

**Fig. 9:**
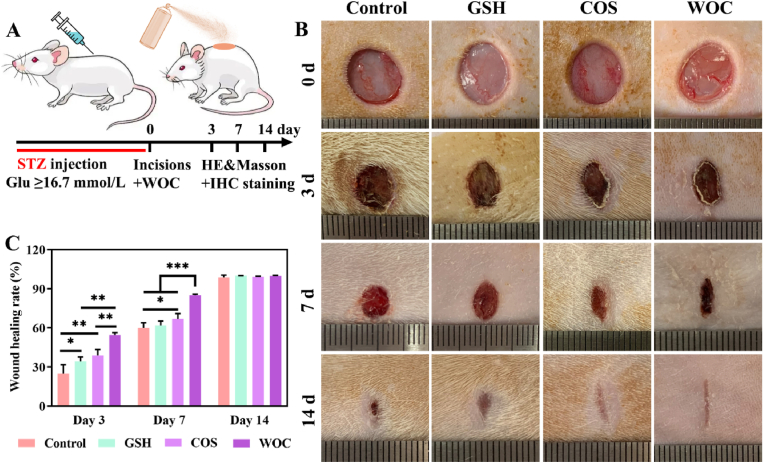
Evaluation of WOC in DW healing. (A) Schematic representation of the streptozotocin-induced diabetic rat model with full-thickness excisional wounds treated via WOC external administration. (B) Representative images of wound healing process. (C) Quantitative assessment of wound healing rate. Data presented as mean ± SD (n = 3). Significance: ∗P < 0.05, ∗∗P < 0.01, ∗∗∗P < 0.001.

The therapeutic potential of WOC for DW repair was evaluated in a streptozotocin-induced diabetic rat model (fasting blood glucose ≥16.7 mmol L^−1^, Fig. 9A) through intravenous administration. Serial photographic documentation revealed distinct wound closure dynamics across treatment groups (Fig. 9B). Control animals showed minimal wound contraction by day 7, with persistent scabbing observable at day 14, indicative of impaired healing. In contrast, GSH-, COS-, and WOC-treated groups demonstrated progressive epithelialization, with WOC exhibiting the most pronounced therapeutic effect. Quantitative analysis confirmed WOC's superior efficacy, achieving 54.57 ± 1.44 % wound closure by day 3 versus 24.83 ± 5.66 % in controls (p < 0.001), and 85.09 ± 0.61 % versus 59.97 ± 3.27 % by day 7 (p < 0.0001) (Fig. 9C).

### 3.6 Histopathological evaluation of DW healing

**Fig. 10:**
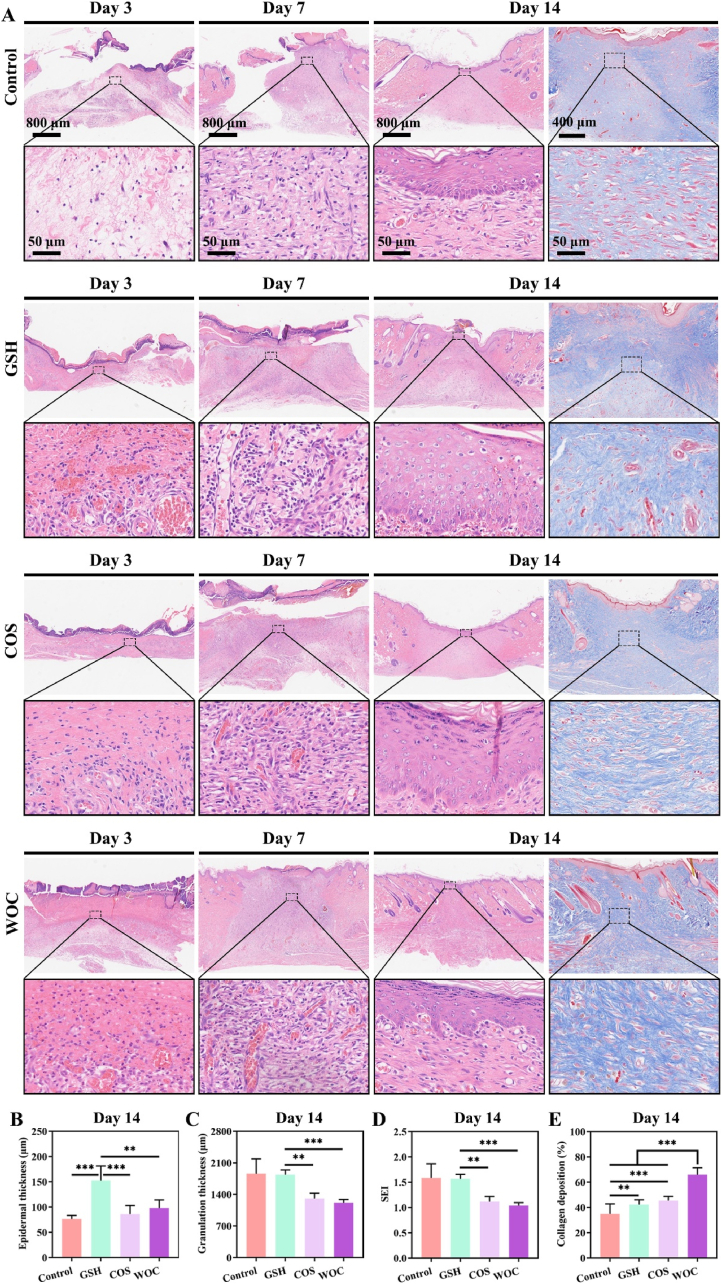
Histopathological and collagen deposition analysis of DW healing. (A) Representative H&E (Red) and Masson's trichrome (blue) staining images. Quantification of (B) epidermal thickness, (C) Granulation thickness, (D) SEI, and (E) collagen deposition (n = 3). Data represent mean ± SD. Statistical significance: ∗∗p < 0.01, ∗∗∗p < 0.001. (For interpretation of the references to color in this figure legend, the reader is referred to the Web version of this article.)

Histopathological analysis (H&E and Masson staining; Fig. 10) revealed distinct healing trajectories across treatment groups. On day 3, the Control group exhibited incomplete tissue ingrowth, while the WOC group demonstrated evident wound closure. By day 7, the Control group still exhibited a substantial wound area without evident scab formation; whereas the WOC group exhibited epidermal regeneration, granulation tissue proliferation, and neovascularization. The regenerative performance of WOC surpassed that of COS and GSH. By day 14, all groups were nearly healed, but differences in epidermal and dermal thickness were observed (Fig. 10A). GSH is a clinically used antioxidant that promotes keratinocyte survival and epidermal regeneration via GSH-Nrf2-thioredoxin cross-talk [53]. The GSH group had significantly thicker epidermis (152.53 ± 24.99 μm) compared to Control (76.29 ± 6.25 μm), COS (86.09 ± 14.72 μm), and WOC (98.04 ± 13.83 μm) (Fig. 10B). Conversely, the WOC group had significantly thinner dermis (1215.47 ± 60.93 μm) than Control (1859.56 ± 284.48 μm), GSH (1837.60 ± 90.42 μm), and COS (1310.49 ± 99.93 μm) (Fig. 10C). Surprisingly, the WOC group had a significantly lower scar elevation index (SEI; 1.04 ± 0.05) compared to Control (1.58 ± 0.24), GSH (1.57 ± 0.08), and COS (1.12 ± 0.09) (Fig. 10D). SEI >1 indicates scar hyperplasia, while SEI = 1 indicates scar-free healing [54]. These findings suggest that GSH repairs DW with excessive scarring, whereas WOC repairs DW with minimal scarring and faster healing. Our previous study showed that metal elements and COS can synergistically inhibit scar hyperplasia, potentially attributed to the elevated anti-inflammatory environment and the down-regulation of ROS/TGF-β1/Smad7 pathway [55].

The deposition and organization of collagen fibrils serve as critical determinants of wound healing quality and functional recovery [56]. Our analysis revealed that WOC orchestrated superior extracellular matrix remodeling, as evidenced by significantly higher collagen density (65.88 ± 5.25 %) compared to control (34.98 ± 7.39 %), GSH (42.27 ± 3.57 %), and COS (45.45 ± 3.12 %) groups at day 14 (p < 0.001; Fig. 10A–E). This enhanced matrix deposition correlated with the characteristic M2 phenotype, which secretes TGF-β and PDGF to activate fibroblasts while maintaining balanced matrix turnover [57].

Notably, while conventional metal-ion/chitosan dressings primarily accelerate healing through granulation and epithelialization [24]. WOC uniquely combined robust collagen synthesis (1.88-fold increase vs Control) with scar suppression (SEI:1.04 vs 1.58 in Control, 1.57 in GSH), demonstrating dual functionality in promoting structural regeneration while preventing pathological fibrosis. This paradigm-shifting outcome suggests WOC's capacity to recalibrate the healing process beyond simple acceleration, achieving both timely closure and optimal tissue remodeling.

### 3.7 Temporal modulation of angiogenesis and inflammatory microenvironment

**Fig. 11:**
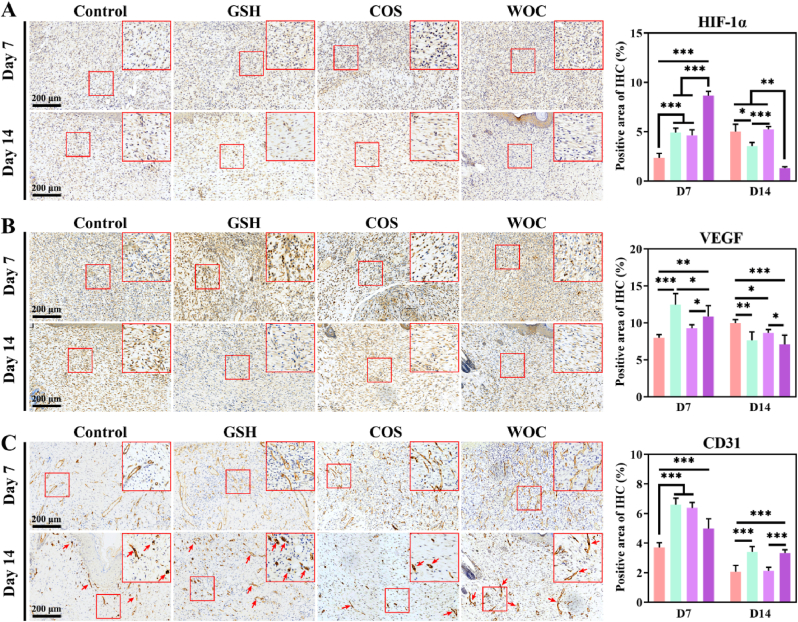
Quantitative analysis of IHC staining (n = 3). (A) HIF-1α; (B) VEGF; (C) CD31. Data represent mean ± SD. Statistical significance: ∗p < 0.05, ∗∗p < 0.01, ∗∗∗p < 0.001.

HIF-1α, VEGF, and CD31 are critical markers of angiogenesis. VEGF is a direct downstream target of HIF-1α, and the HIF-1α/VEGF pathway can be directly driven to stimulate angiogenesis by hypoxia, agents, and biomaterials [[58], [59], [60]]. IHC analysis revealed that WOC treatment significantly upregulated HIF-1α and VEGF expression during the early proliferative phase (day 7), with positive staining areas increasing by 3.69-fold (p < 0.001) and 1.36-fold (p < 0.01) respectively compared to controls, indicating robust activation of hypoxia-driven angiogenesis. By day 14 (maturation phase), these markers decreased to baseline levels, reflecting the resolution of hypoxic stress as vascularization neared completion (Fig. 11A–B). In contrast, CD31^+^ endothelial cell density remained elevated in WOC-treated wounds at both time points (day 7: 1.35-fold; day 14: 1.61-fold vs control, p < 0.001), demonstrating sustained vascular network maturation (Fig. 11C, red arrows). This biphasic regulation - rapid induction followed by timely resolution of angiogenic stimuli - coupled with persistent CD31 expression suggests WOC uniquely coordinates both the initiation and stabilization phases of neovascularization. The sustained CD31 signal may further reflect enhanced endothelial-immune cell crosstalk, potentially contributing to the observed anti-inflammatory microenvironment and superior healing outcomes [61].

**Fig. 12:**
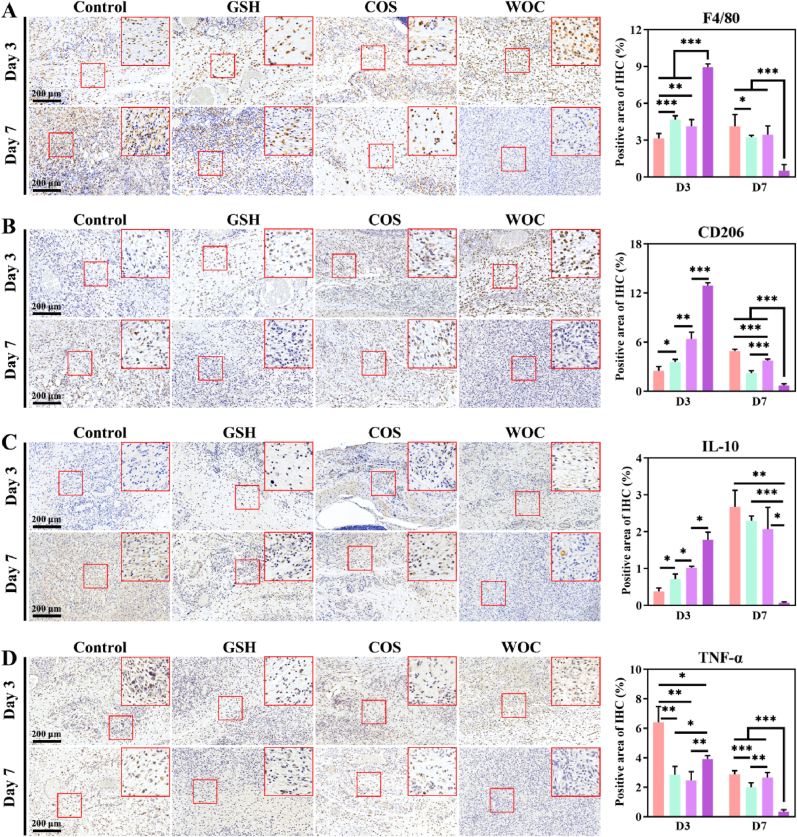
Quantitative analysis of IHC staining (n = 3). (A) F4/80; (B) CD206; (C) IL-10; (D) TNF-α. Data represent mean ± SD. Statistical significance: ∗p < 0.05, ∗∗p < 0.01, ∗∗∗p < 0.001.

IHC analysis of wound tissues revealed distinct temporal patterns in macrophage recruitment and polarization. At day 3 post-injury, WOC-treated wounds exhibited a 2.84-fold increase in F4/80^+^ macrophage infiltration (p < 0.001) and a 5.16-fold elevation in CD206^+^ M2 macrophages (p < 0.001) compared to controls, demonstrating rapid immune cell recruitment and polarization (Fig. 12A–B). This early M2 dominance correlated with enhanced anti-inflammatory signaling, as evidenced by a 4.66-fold increase in IL-10 expression (p < 0.001) and a 38.76 % reduction in TNF-α levels (p < 0.001) versus controls (Fig. 12C–D), establishing an optimal pro-healing microenvironment. By day 7, as wound repair progressed through inflammation resolution and programmed macrophage apoptosis [62], all inflammatory markers in WOC-treated wounds returned to baseline levels significantly faster than other groups. This precise temporal regulation - rapid immune activation followed by timely resolution - contrasts with the prolonged inflammation observed in controls (8.42-fold higher TNF-α at day 7, p < 0.001) (Fig. 12D) and demonstrates WOC's unique capacity to orchestrate the complete healing cascade from inflammatory initiation to resolution.

**Fig. 13:**
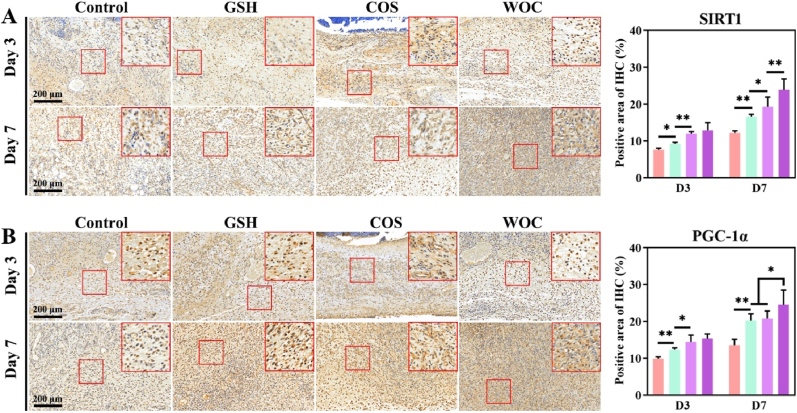
Quantitative analysis of IHC staining (n = 3). (A) SIRT1, and (B) PGC-1α. Data represent mean ± SD. Statistical significance: ∗p < 0.05, ∗∗p < 0.01.

There was a sustained upregulation of key mitochondrial biogenesis regulators (SIRT1 and PGC-1α) in WOC-treated wounds at both day 3 and day 7 post-injury (Fig. 13A–B). SIRT1 and PGC-1α orchestrate metabolic shift toward OXPHOS, yielding two critical therapeutic benefits: (1) generation of anti-inflammatory mediators through enhanced mitochondrial respiration, and (2) maintenance of redox homeostasis [63]. Notably, the persistent elevation of SIRT1/PGC-1α through day 7 suggests their additional role in supporting the later stages of tissue remodeling, despite inflammation resolution and macrophage apoptosis. This prolonged activation of mitochondrial biogenesis pathways likely contributes to WOC's superior healing outcomes by coordinating both the inflammatory resolution and regenerative phases of wound repair.

## 4 Conclusion

In this study, we engineered a powerful and smart WOC nanoplatform via a simple one-pot method to achieve adaptive healing of DW. While there are many methods to reduce inflammation and promote angiogenesis, our WOC nanoplatform delivers a triple-axis therapeutic strategy that uniquely: (1) Synchronizes microenvironment sensing with cellular reprogramming. WOC's glucose/pH/ROS-responsiveness enables adaptive drug release in DWs, outperforming pH-only or glucose-sensitive carriers; Colorimetric transition provides real-time treatment feedback—unmatched by passive dressings. (2) Resolves inflammation via mitochondrial bioenergetics (not cytokine blockade). WOC drives M2 polarization through OXPHOS amplification, avoiding the immunosuppression risks of anti-inflammatory cytokine delivery; By enhancing mitochondrial biogenesis, WOC sustains M2 commitment without repeated dosing. (3) Restores angiogenesis via intercellular mitochondrial transfer (not growth factors). Dynamin-dependent mitochondrial transfer rescues endothelial bioenergetics, enabling physiological vascular repair without recombinant VEGF risks; Simultaneously reprograms macrophages (donors) and endothelium (recipients). (4) Achieves scarless healing through matrix remodeling. WOC balances synthesis and alignment, surpassing GSH and collagen scaffolds that induce fibrosis.

## CRediT authorship contribution statement

**Xiuhong Huang:** Writing – review & editing, Writing – original draft, Validation, Methodology, Investigation, Data curation, Conceptualization. **Ziling Lin:** Validation, Supervision, Project administration, Funding acquisition. **Mingshu Ruan:** Investigation, Formal analysis, Data curation. **Peizhen Huang:** Resources, Investigation, Funding acquisition, Formal analysis. **Hongmei Ding:** Resources, Methodology, Formal analysis. **Hao Pan:** Resources, Methodology, Formal analysis. **Jiahui Cao:** Resources, Methodology, Formal analysis. **Chunmei Ma:** Resources, Methodology, Formal analysis. **Qianhao Zhao:** Resources, Methodology, Formal analysis. **Wenping Guo:** Resources, Methodology, Formal analysis. **Keke Wu:** Writing – review & editing, Validation, Supervision. **Chongkai Fang:** Writing – review & editing, Supervision, Methodology, Investigation. **Aijun Liu:** Writing – review & editing, Validation, Supervision. **Liqin Zheng:** Writing – review & editing, Writing – original draft, Supervision, Methodology, Investigation, Funding acquisition, Conceptualization.

## Funding

This work was supported by 10.13039/501100001809National Natural Science Foundation of China (82374469); Scientific research project of 10.13039/501100010883Traditional Chinese Medicine Bureau of Guangdong Province, Special research platform (20254022); University-Hospital Joint Fund Project of Guangzhou University of Chinese Medicine-Shenzhen Hospital Fund (GZYFT2024Y02); National Research Project of the Center for Inheritance and Innovation of Traditional Chinese Medicine (2022QN10); 2025 High-Level Talent Aggregation Project Research Funding (A1-2601-25-414-108Z06); 2025 "Summit Pioneering" Action Plan - "Foundation Strengthening" Program for First-Class Discipline Capacity Enhancement (A1-2601-25-415-108Z427).

## Declaration of competing interest

The authors declare that they have no known competing financial interests or personal relationships that could have appeared to influence the work reported in this paper.

## Data Availability

Data will be made available on request.
