# Supplementary material for: Light up the mitochondria: Smart tungstate-oligosaccharide nanoplatform orchestrates mitochondrial transfer from M2 macrophages to restore endothelial function for adaptive diabetic wound regeneration
Source: Mater Today Bio. 2025 Aug 14;34:102196. doi: 10.1016/j.mtbio.2025.102196 (PMC12391290; doi:10.1016/j.mtbio.2025.102196)
Supplement: Multimedia component 1 [file mmc1.docx]

# Light up the mitochondria: Smart tungstate-oligosaccharide nanoplatform orchestrates mitochondrial transfer from M2 macrophages to restore endothelial function for adaptive diabetic wound regeneration

Xiuhong Huang ^a^, Ziling Lin ^b^, Mingshu Ruan ^c^, Peizhen Huang ^b^, Hongmei Ding ^b^, Hao Pan ^a^, Jiahui Cao ^a^, Chunmei Ma ^a^, Qianhao Zhao ^a^, Wenping Guo ^a^, Keke Wu ^d, *^, Chongkai Fang ^e, *^, Aijun Liu ^a, *^, Liqin Zheng ^b, *^

^a^ School of Basic Medical Sciences, Guangzhou University of Chinese Medicine, Guangzhou 510006, China

^b^ Department of Hand Surgery and Wound Repair, The First Affiliated Hospital of Guangzhou University of Chinese Medicine, Guangzhou 510405, China

^c^ International Collage, Guangzhou University of Chinese Medicine, Guangzhou 510405, China

^d^ School of Biomedical Engineering, Affiliated Cancer Hospital & Institute, Guangzhou Medical University, Guangzhou 511436, China

^e^ Science and Technology Innovation Center, Guangzhou University of Chinese Medicine, Guangzhou 510405, China

*Corresponding authors:

Dr. Liqin Zheng (ucmlykhin@stu.gzucm.edu.cn)

Prof. Aijun Liu (aijunliu@gzucm.edu.cn)

Dr. Chongkai Fang (fang.chongkai@hotmail.com)

Prof. Keke Wu (drwukeke@126.com)


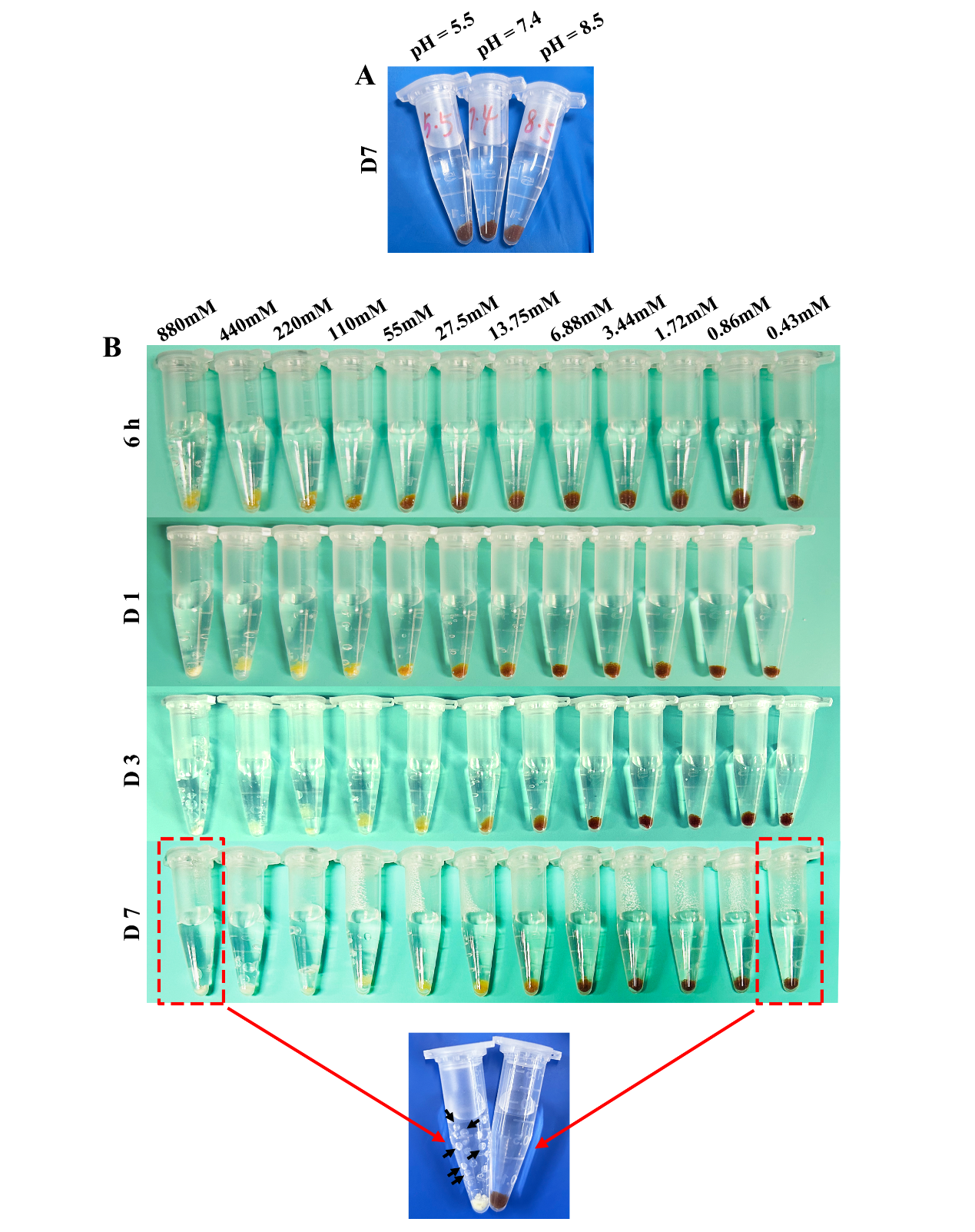


**Fig. S1**. A concomitant visual transition in WOC’s colorimetric profile correlated with H_2_O_2_ concentration and scavenging but pH value.


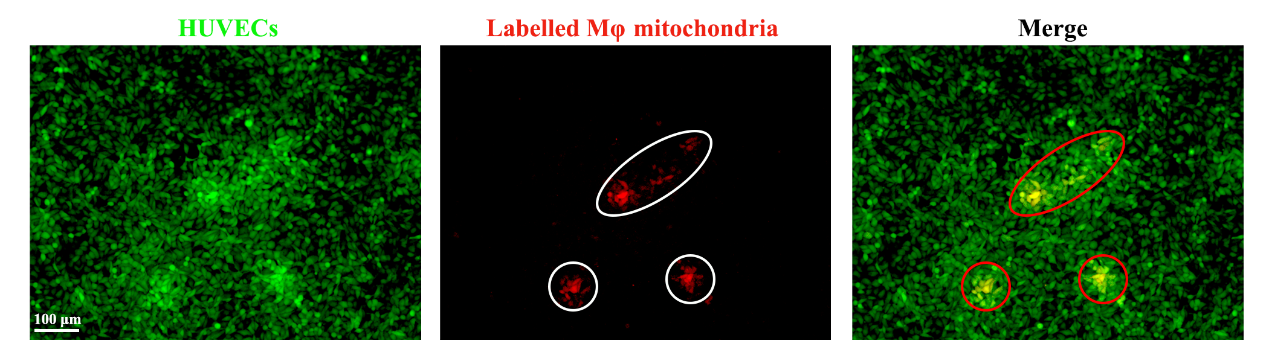


**Fig. S2**. HUVECs exhibit mitochondrial tropism.


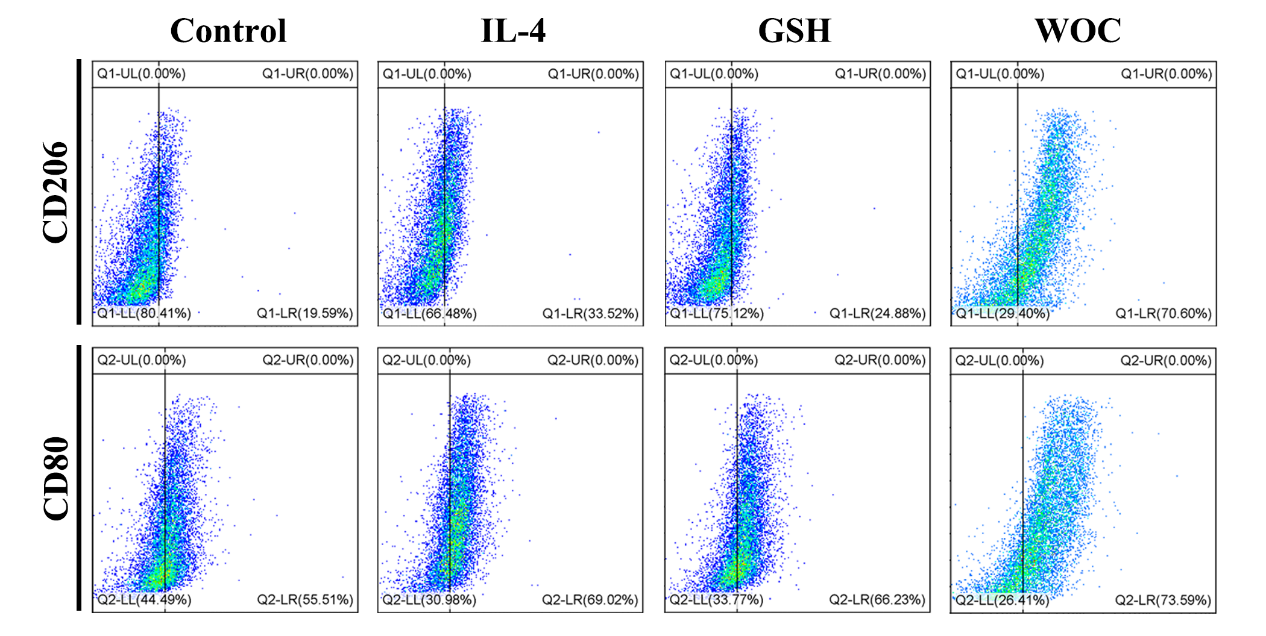


**Fig. S3**. Flow cytometry revealed a significant increase in CD206/CD80 ratio of macrophages under WOC treatment.
